# 

**Published:** 1978

**Authors:** 


JULY/OCTOBER 1978

VOLUME 93 (iii/iv)

NUMBER 347/348

Published Quarterly

Annual Subscription £6 Post Free

### BRISTOL MEDICO-CHIRURGICAL JOURNAL

Contents

3 Porphyrias and Mental Handicap

J. Jancar

7 Associations between HLA Antigens and Disease

Geoffrey H. Tovey

Established 1883

### BRISTOL MEDICO-CHIRURGICAL JOURNAL

Incorporating the Medical Journal of the South West

### Editorial Committee

EDITOR
*Dr. D. S. Reeves*
*Southmead Hospital, Bristol, BS10 5NB*
ASSISTANT EDITOR
*Dr. A. P. Radford*
MEMBERS
*Dr. Rhys Davies*
*Dr. M. B. Lennard*
*Dr. D. W. Wright*
*The Hon. Treasurer of the Society*
*The Hon. Secretary of the Society*
SECRETARY
*Mr. B. P. Jones, The Library, Medical School, University Walk, Bristol, BS8 1TD*

### Bristol Medico-Chirurgical Society

PRESIDENT 1978/79
*H. K. Lucas, Esq.*
PRESIDENT ELECT
*Dr. D. S. Ractliffe*
HONORARY SECRETARY
*A. J. Webb, Esq., 7 Percival Road, Bristol, BS8 3LE*
HONORARY TREASURER
*Dr. J. Penry, Southmead Hospital, Bristol, BS10 5NB*

The Society was founded in 1874. In 1883 publication of the Journal began and has continued quarterly ever since then. During the years 1949 to 1962 it appeared under the title of 'The Medical Journal of the South West'.

### Notice to Contributors

*The Journal is especially concerned to provide a place for the recording of medical thought, interests, and practice in and around Bristol. It therefore welcomes articles that, as well as being of immediate interest to members, will document the local and contemporary medical scene for our successors.*

*Original articles are invited provided that they have not been published elsewhere. Illustrations should be supplied ready for reproduction. Line drawings should be in Indian ink on firm white paper or board. The author will be informed if it is found necessary to ask him to pay for the printing of photographs etc.*

*The following contributions will be especially welcome; notes of forthcoming events, meetings held, degrees conferred, staff appointments, honours and public appointments and other local news.*

*Advice on preparation can be obtained from the Editor.*

Printed by the University of Bristol Printing Unit

### University of Bristol

### FACULTY OF MEDICINE

**Final Examination for the Degrees of M.B., Ch.B.**
**June 1978**

### PASS WITH HONOURS

Mark Henry BEER
with Distinction in Pathology
Barbara Anne BROWN
with Distinctions in Community Health and in Mental Health
David Ian COOKE
with Distinctions in Obstetrics & Gynaecology and in Pathology
Robin Frank GRIFFITHS
with Distinctions in Surgery and in Child Health
Carol Ann NAUGHTON
with Distinction in Obstetrics & Gynaecology
Christopher James Irving PAYNE
with Distinction in Community Health

### PASS

Anne ALEXANDER
Mervyn Bromley AMESBURY
Peter ARMSTRONG
Wendy Elizabeth BARBER
with Distinction in Mental Health
Peter Michael BARROW
Jonathan Martin BELL
Sylvia Irene BERNEY
Nicholas Meirion BIRCHALL
Christopher Richard BOWMAN
Jennifer Wanda BROWN
David Wayne BULLOCK
Kenneth Rodney BURCHETT
Steven Richard BURNLEY
David Stirling CHESNEY
John CHURCH
Andrew William COE
Kenneth John COLEMAN
Stephen Leonard COLLIER
James Donald COLLINS
Robert George DALTON
Angela Margaret DAVIES
Graham John DEAKIN
Richard DOYLE
Jennie Mair EDWARDS
Alun Cameron ELIAS-JONES
Esmail Anverali ESMAILJI
Christopher James EVERED
John Barrie FIRTH
Mary Geraldine FLYNN
David Martin FOREMAN
Rosemary Jill FULTON
Gerard Harry Raleigh GIBBS
Robert William GIBBS
Julia Mary Jean GIFFORD
Christopher Douglas GOOD
David Thomas GOODRUN
David Brian GRIFFITH
Peter John HALDEN
Granville Joseph HANCOCK
Geraldine HARRIS
Sarah Jane HARRIS
Helen HAWKRIDGE
Penelope Jane HENLEY
Alison HERDMAN
Lietta Caron HEUFELD
Andrew Sutherland HORNE
John Howard INGLE
Hamidah KARIM
Norah Christine Joan KILLEEN
David Graham KINGDON
Jane Elizabeth LANGRIDGE-SMITH
Michael Joseph LEONARD
Piers Julian Anthony LESSER
Richard Julian LEVITT
Christopher Edward George MARTIN
Ian Douglas McLAREN
Dhirendra MEHTA
Sheila Mary MONTGOMERY
Susan Charlotte MOSER
Margaret MURPHY
James William NEAL
Katherine NICHOLLS
Jeremy John NIGHTINGALE
Michael O'CONNOR
Anthony John O'REILLY
Brian Joseph O'REILLY
Rebecca Jane O'REILLY
Glynis PARKER
Dilip Kumar Kunverji PATEL
Nikunjkumar Ishwarbhai PATEL
Stephen John PEDLAR
Phillip Edward PEMBERTON
Colin John PHILIP

John QUICKE
Jonathan Robert Spencer RAMSAY
Jonathan Nicholas RICHARDS
Fiona Anne RITCHIE
Robert Stephen RIVETT
Frederick Leighton ROBERTS
Susan Mary ROBERTS
Jonathan Charles ROGERS
Jacqueline Clare ROSSITER
Martin Neal ROWAN-ROBINSON
Peter Bertram RUMBLE
Richard Emsley SARGINSON
Robert Baliol SCOTT
Stephen Andrew SMITH
Susan Margaret STANTON
Steven Richard STERN
Christine June SULLIVAN
David Geoffrey Barrie TAYLOR
Janet Mary THOMAS
Alvan Harold TROUGHTON
Carol Suzanne TWEED
Habib ULLAH
Stephen Andrew VERNON
Lisbeth Lynda WATT
Mary Annette WELSH
Pamela Jean WHITBY
David Eric WILKINSON
Marion Siobhan WILLIAMS
James WILSON-MACDONALD
Caroline Jane YOUNG

**Final Examination for the Degree of B.V.Sc.**
**June 1978**

**PASS WITH HONOURS**

Katrine Jane BERRY
with Credit in Veterinary Medicine
Neil CRAVEN
with Credits in Veterinary Medicine and in Veterinary Surgery
Richard Peter HIND
Michael Bruno French NATHAN
Gordon John OXENHAM
Simon Kyffin SHANKLIN
with Credit in Veterinary Medicine

**PASS**

Roger Hugh ALISON
Nicholas John BURDEN
Charles Frank COLEMAN
Peter John CRIPPS
Ann Margaret DEAKIN
Peter Graham DING
Michael John DUNCALFE
Christine Jane GRADWELL
Christopher John HANSON
Jonathan James HOLT
Elizabeth Davina Morgan JONES
Geoffrey KERSHAW
Elizabeth Kendal KIRKLAND
Charles Martin LOWE
Simon MADDY
Colin Adrian ROBERTS
Carol Anne SHENTON
Patricia SHORT
Jennifer SMITH
Gary STEVENS
Jane Morag THOMSON
Joanna Clare TURNER
Marjorie Helen VENABLES
Guy Berkeley WARDLE
Anthony Joseph WEBB
with Credits in Veterinary Medicine and in Veterinary Surgery
Jacqueline WEIR
with Credit in Veterinary Surgery
Huw Rhys WILLIAMS

**September 1978**

Vanessa FOULDS
Susan Christina HOARE

**Doctor of Medicine**
**June 1978**

Anthony Edward Buckland GIDDINGS

**October 1978**

Peter Graham BAKER

**Doctor of Philosophy**
**June 1978**

Bernard John MOXHAM
Alistair Ian WEBB

**Master of Science in Medical Sciences**
**June 1978**

Mohamed Thahir Mohamed JIFFRY
Razmik MINASSIAN

**October 1978**

John Frederick HUNTLEY
Atilio Alberto YANEZ

**Master of Dental Surgery**
**October 1978**

Christopher David STEPHENS

### UNIVERSITY OF BRISTOL MEDICAL LIBRARY SELECTED NEW BOOKS — DECEMBER 1978

These are books acquired — not necessarily bought and not necessarily fully processed — in December. All are available for borrowing now.

Advances in Protein Chemistry. Vol. 32 (1978). Academic.

ALLFREY, V. G. et al. Organisation and expression of chromosomes. Dahlem Konferenzen 1976.

ALTMAN, A. and SCHWARTZ, A. D. Malignant diseases of infancy, childhood and adolescence (Major problems in clinical pediatrics) Saunders.

ASBURY, A. K. and JOHNSON, P. C. Pathology of peripheral nerve. Saunders.

BAGGOT, J. D. Principles of drug disposition in domestic animals: the basis of veterinary clinical pharmacology. Saunders.

Barefoot Doctor's Manual. Revd. and enlarged ed. prepared by the Revolutionary Health Committee of Human Province. Routledge.

BARUCH, G. and TREACHER, A. Psychiatry observed. Routledge.

BASMAJIAN, J. V. Surface anatomy: an instruction manual. Williams & Wilkins.

BECKER, F. Cancer: a comprehensive treatise. Vols. 3, 5, 6. (Vols. 1, 2, 4 already in stock). Plenum.

BEE, H. The developing child. Ed. 2. Harper & Row.

BELLANTI, J. A. Immunology 2. Saunders.

BERLYNE, G. M. A course in renal diseases. Ed. 5. Blackwell.

BERRY, P. Language and communication in the mentally handicapped. Univ.Park Pr.

BIRZLE, H. et al. Radiology of trauma. Saunders.

BLECHER, M. Methods in receptor research (Methods in molecular biology, Vol. 9, Pts. 1—2) 2 Vols. Dekker.

BOCHNER, F. et al. Handbook of clinical pharmacology. Little, Brown.

BOLOGNESE, R. J. and SCHWARZ, R. H. Perinatal medicine: clinical management of the high-risk fetus and neonate. Williams & Wilkins.

BORRIE, J. Management of emergencies in thoracic surgery. Ed. 2. ACC 1972.

BOSZORMENYI-NAGY, I. and SPARK, G. M. Invisible loyalties. Harper & Row. 1973.

BOWEN, W. H. et al. Immunologic aspects of dental caries (Special suppl. to 'Immunology abstracts') Information Retrieval Inc.

BRANDER, G. C. and PUGH, D. M. Veterinary applied pharmacology and therapeutics. Ed. 3. Bailliere.

BROWN, R. G. S. The changing National Health Service. Ed. 2. Routledge.

BRUMFITT, W. New perspectives in clinical microbiology, 1. Kluwer.

BRYANT, N. J. Laboratory immunology and serology. Saunders.

BUSCH, H. The cell nucleus, Vols. 4—5. Academic.

BUTLER, P. M. and JOYSEY, K. A. Development, function and evolution of teeth. Academic.

CAPALDI, R. A. Membrane proteins and their interactions with lipids. Dekker.

CASS, J. et al. Laboratory animals: an annotated bibliography. Hafner. 1971.

CLARKE, E. G. C. and CLARKE, M. L. Veterinary toxicology (previously Garner's 1975) repr. Bailliere. 1978.

Clinical Orthopaedics and Related Research. Vol. 135 (Sept. 1978) Lippincott.

Clinics in Haematology, 7:3 (October 1978): Aplastic anaemia. Saunders.

Clinics in Obstetrics and Gynaecology. 5:3 (Dec. 1978): Gynaecological Surgery. Saunders.

Clinics in Perinatology. 5:2 (Sept. 1978): The respiratory system. Saunders.

CLUFF, L. E. and JOHNSON, J. E. Clinical concepts of infectious diseases. Ed. 2. Williams & Wilkins.

COLLINS, R. D. Illustrated diagnosis of localised diseases. Lippincott. 1974.

COMBE, A. Observations on mental derangement ... facsim. reprod. of 1st American ed. (1834) Scholars' Facsimiles & Reprints Delmar, N.Y. 1972.

CONN, H. F. Current therapy 1978. Saunders.

COURTOIS, Y. and REGNAULD, F. Colloque: biology of the epithelial lens cells in relation to development, ageing and cataract. INSERM 1976.

CRAMER, H. and SCHULTZ, J. Cyclic 3',5'-nucleotides: mechanisms of action. Wiley.

CRANDALL, B. F. and BRAZIER, M. A. B. Prevention of neural tube defects: the role of alpha-fetoprotein. Academic.

DONINI, I. and BATTEZZATI, M. The lymphatic system. Translated by V. Cameron-Curry. Piccin 1972.

DRILLEN, C. M. and DRUMMOND, M. B. Neurodevelopmental problems in early childhood: assessment and management. Blackwell.

DUNCKER, H. R. The lung air sac system of birds. (Advances in anatomy) Springer 1971.

EASTHAM, R. D. Biochemical values in clinical medicine. Ed. 6. Wright.

EVANS, E. F. and WILSON, J. P. Psychophysics and physiology of hearing. Academic.

EVERETT, D. H. and VINCENT, B. Ions in macromolecular and biological systems (Colston papers, 29). Scientechnica.

FESTENSTEIN, H. and DEMANT, P. HLA and H—2 — basic immunogenetics, biology and clinical relevance. Arnold.

FOX, S. A. Lid surgery: current concepts. Grune & Stratton 1972.

FRANCOIS, J. and RYSSELAERE, M. Oculomycoses. C.C. Thomas 1972.

FROMM, D. Complications of gastric surgery. Wiley.

FRY, J. A new approach to medicine: principles and priorities in health care. MTP.

FUDENBERG, H. H. et al. Basic and clinical immunology. Ed. 2. Lange.

GALLO, R. C. Cancer research: cell biology, molecular biology and tumour virology. CRC Press. 2 Vols.

GASKING, E. B. Investigation into generation, 1651—1828. Hutchinson 1967.

GAZZANIGA, M. S. and BLAKEMORE, C. Handbook of psychobiology. Academic.

General Board of Health. Papers relating to the sanitary state of the people of England: being the results of an inquiry into the different proportions of death produced by certain diseases in different districts in England. HMSO 1858.

GERSHON, S. and SHOPSIN, B. Lithium: its role in psychiatric research and treatment. Plenum Press. 1973 repr. 1976.

GIRARDIER, L. and SEYDOUX, J. Effectors of thermogenesis (Experientia Suppl. 32) Birkhauser Vlg. Basel.

Gloucester. Health Committee. Reports as to the epidemic of mild small-pox in 1923. Smart 1923.

GOODLAND, N. L. General intensive care. Wright.

GORDON, G. Active touch. Pergamon.

GOTTLIEB, G. Studies on the development of behaviour and the nervous system. Vol. 4: Early influences. Academic.

GURMAN, A. S. and RAZIN, A. M. Effective psychotherapy: a handbook of research. Pergamon.

HALL, J. L. and BAKER, D. A. Cell membranes and ion transport. Longman.

Handbook of Lipid Research, (ed.) D. J. Hanaham. Vol. 1: Fatty acids and glycerides, (ed.) A. Kuksis. Vol. 2: The fat-soluble vitamins, (ed.) H. F. DeLuca. Plenum.

HANSHAW, J. B. and DUDGEON, J. A. Viral diseases of the fetus and newborn (Major problems in clinical pediatrics) Saunders.

HARRISON, I. E. and COSMINSKY, S. Traditional medicine: an annotated bibliography of Africa, Latin America and the Caribbean. Garland STRM Press.

HEATH, G. The illusory freedom: the intellectual origins and social consequences of the sexual 'revolution'. Heinemann.

HEMMINGS, W. A. Antigen absorption by the gut. MTP.

HISCOCK, I. V. Community health organisation. Ed. 4. Commonwealth Fund 1950.

Inter-departmental Committee on Abortion. Report. HMSO 1939.

International Congress for the History of Medicine, 24th. Acta congressus internationalis XXIV historiae artis medicinae. Kultura. 2 Vols.

ISAAC, G. L. and McCOWN, E. R. Human origins: Louis Leakey and the East African evidence. W. A. Benjamin 1976.

JELJASZEWICZ, J. Staphylococci and staphylococcal diseases. Fischer 1976.

JONES, E. S. Essential intensive care. MTP.

JONES, F. A. et al. Peptic ulcer healing: recent studies on carbenoxolone. MTP.

KALEY, G. and ALTURA, B. M. Microcirculation. Vol. 2. Univ.Park Pr.

KATZ, D. H. Lymphocyte differentiation, recognition and regulation. Academic.

KEERS, R. Y. Pulmonary tuberculosis: a journey down the centuries. Bailliere.

KOREN, J. The history of statistics. Burt Franklin 1918 repr. 1970.

KOSTYUK, P. G. Structure and function of descending systems of the spinal cord. Transl. from Russian. IPST/Keter.

KUCHLER, R. J. Biochemical methods in cell culture and virology. Dowden.

LEE, J. and LAYCOCK, J. F. Essential endocrinology. Oxford UP.

LEEUWENBERG, E. L. J. and BUFFART, H. F. J. M. Formal theories of visual perception. Wiley.

LEIGH, D. et al. A concise encyclopaedia of psychiatry. MTP.

LEWIS, J. G. Guide to house physicians in the medical unit. Ed. 3. Heinemann.

LINDHEIMER, M. D. et al. Hypertension in pregnancy. Wiley.

LIPP, M. R. Respectful treatment: the human side of medical care. Harper & Row.

LUNDBLAD, R. L. et al. Chemistry and biology of thrombin. Ann Arbor Science Publ.

McCOLLUM, E. V. The newer knowledge of nutrition. Ed. 2. Macmillan 1922.

MacGREGOR, A. Public health in Glasgow, 1905–1946. Livingstone 1967.

MACKINTOSH, J. M. Trends of opinion about the public health, 1901–51. Oxford UP. 1953.

MACKINTOSH, J. M. The War and mental health in England. Commonwealth Fund 1944.

McNICHOLL, B. et al. Perspectives in coeliac disease. MTP.

MAHY, B. W. J. and BARRY, R. D. Negative strand viruses and the host cell. Academic.

MESCHAN, I. Radiographic positioning and related anatomy. Ed. 2. Saunders.

Methods in Microbiology, (eds.) T. Bergan and J. R. Norris. Vol. 10. Academic.

MEYNELL, G. G. and MEYNELL, E. Theory and practice in experimental bacteriology. Ed. 2. Cambridge UP.

MINUCHIN, S. et al. Psychosomatic families: anorexia nervosa in context. Harvard UP.

MITCHELL, J. P. et al. A handbook of surgical diathermy. Ed. 2. Wright.

MUSTARD, H. S. Rural health practice. Commonwealth Fund 1936.

NAISH, J. M. et al. The clinical apprentices. Ed. 5. Wright.

National Association for the Promotion of Social Science, Annual Congress, 7th Edinburgh, 1863. Transactions. Longman 1864.

National Association for the Promotion of Social Science. Annual Congress, 13th, Bristol, 1869. Transactions. Longman 1870.

**SPECIMEN COPIES RECEIVED OF JOURNALS NOT SUBSCRIBED TO**

Anatomian Clinica 1:1 (1978)
Angiology 29:1 (Jan. 1978)
Chest, Heart & Stroke Journal 2:4 (Winter 1977/78)
Child Abuse & Neglect 2:1 (1978)
Current Microbiology 1:1 (1978)
Hearing Research 1:1 (Oct. 1978)
Journal of Computer-assisted Tomography 2:5 (1978)
Mental Handicap Bulletin 26 (Sept. 1977)

**SOME NEW JOURNALS ANNOUNCED**

Biopharmaceutics & Drug Disposition. Wiley.

Clinical & Laboratory Haematology. 1:1 (Mar. 1979). Blackwell.

Parasite Immunology. 1:1 (Jan. 1979). Blackwell.

**DISCONTINUED GIFTS**

After 1978, the Library will no longer receive as gifts the Journal of International Medical Research and the Medico-Legal Journal. If anyone is prepared to part with 1979 issues, the Librarian would be grateful to receive them.

**CHANGE OF TITLE**

The Proceedings of the Society for General Microbiology have now become the Society for General Microbiology Quarterly.

